# Modulation of FOXP3 Gene Expression in OVCAR3 Cells Following Rosmarinic Acid and Doxorubicin Exposure

**DOI:** 10.3390/ph17121606

**Published:** 2024-11-28

**Authors:** Veysel Toprak, İlhan Özdemir, Şamil Öztürk, Orhan Yanar, Yusuf Ziya Kizildemir, Mehmet Cudi Tuncer

**Affiliations:** 1 Private Metrolife Hospital, Şanlıurfa 63320, Turkey; drveysel21@outlook.com; 2 Department of Gynecology and Obstetrics, Faculty of Medicine, Atatürk University, Erzurum 25070, Turkey; ilhanozdemir25@yandex.com; 3 Vocational School of Health Services, Çanakkale Onsekiz Mart University, Çanakkale 17100, Turkey; ozturksamil@outlook.com; 4 Private Nev Hospital, Şanlıurfa 63300, Turkey; drorhan6532@outlook.com; 5 Şanlıurfa Training and Research Hospital, Şanlıurfa 63300, Turkey; dryusufziya34@outlook.com; 6 Department of Anatomy, Faculty of Medicine, Dicle University, Diyarbakir 21200, Turkey

**Keywords:** ovarian cancer, FOXP3, apoptosis, rosmarinic acid, tumor suppressor

## Abstract

**Background/Objectives:** Ovarian cancer has the highest mortality rate in the world. Treatment methods are listed as surgery, chemotherapy, and radiotherapy, depending on the stage of cancer, but developing resistance to chemotherapy increases the need for alternative agents that act on the same pathways. The effects of rosmarinic acid (RA) and doxorubicin (DX) on the activation of FOXP3, an important tumor suppressor gene, in OVCAR3 cells were examined. **Materials and Methods:** In this study, a human ovarian adenocarcinoma cell line was used. MTT analysis was performed to reveal the result of RA and DX on ovarian cancer cell proliferation. Expression levels of FOXP3 for cell proliferation and Capase-3 for apoptosis were determined by RT-qPCR. The wound healing model was applied to determine cell migration rates. The results were evaluated with one-way ANOVA in an SPSS 20.0 program as *p* ≤ 0.05. **Results:** It was determined that RA and DX alone and in combination inhibited the proliferation of OVCAR3 cells in different doses for 24, 48, and 72 h, and caused the cells to die by causing them to undergo apoptosis. Caspase-3 expression increased approximately tenfold in OVCAR3 cells, while FOXP3 expression was upregulated only in RA treatment and was downregulated in DX and RA + DX treatments. **Conclusions:** According to the results of our study, it was determined that the FOXP3 signaling pathway related to apoptosis, and proliferation was affected by the combination treatment of RA and DX in the OVCAR3 cancer cell line. This shows that RA will gain an important place in cancer treatment with more comprehensive study.

## 1. Introduction

Among the diseases that most affect women in the world, ovarian cancer ranks third in terms of malignancy. However, it ranks first among cancers in terms of mortality rate. Moreover, its non-symptomatic course, late diagnosis, and frequent recurrence are other disadvantages [1,2,3]. When diagnosed, peritoneal involvement and intraperitoneal metastasis usually occur together [2,4]. Ovarian cancer cells cause relapses to occur more frequently because of the resistance they develop to chemotherapy [5]. Although the characteristic etiology of ovarian cancer is unknown, since the clinical manifestations of these cancers occur in advanced stages, the molecular mechanisms that define or reveal its development and progression are unclear.

Today, plants preferred for medicinal purposes have become important not only for drug development, but also as food supplements. RA is widely compounded in many plants and is a powerful polyphenol. Among the plants where it is found in the highest concentration are rosemary and sage. It is one of the components that give these plants their aromatic properties [6]. RA exhibits very strong antioxidant properties, and this feature is more effective than vitamin E. While RA reduces the risk of chronic diseases such as cancer, it reduces free radical-induced cell damage [7]. In an in vivo study conducted with various different doses of RA, they investigated its mutagenicity against the antitumoral agent DX using the micronucleus method in the blood cells of mice. Those treated with RA showed antioxidant capacities with results close to the control. A significant increase in micronucleus frequency was detected in subjects given DX and RA in combination. Although the mechanism explaining the protective effect is unknown, it has been reported that RA also exhibits potent antioxidant against DX toxicity [8].

The master transcription factor forkhead box protein P3 (FOXP3) characterizes nTregs produced after the activation of naive T cells in the presence of TGFβ [9]. FOXP3 is a member of the forkhead helix family of transcription factors, a gene family that plays a central role in the function of the most widely recognized and best-studied subsets of immune system regulators: T cells [10]. Tregs work to suppress an autoimmune response in the body, and in cancer this action allows cancerous cells to escape the antitumor response and can suppress antitumor immunity [11,12].

In this study, we aim to investigate the modulation of FOXP3 gene expression in OVCAR3 cells following treatment with rosmarinic acid and doxorubicin. Understanding the role of FOXP3, a crucial regulator of immune tolerance, in the context of ovarian cancer cells could offer valuable insights into its potential as a therapeutic target. By examining the combined effects of rosmarinic acid and doxorubicin, we seek to explore novel strategies to enhance cancer cell sensitivity to chemotherapy and potentially reduce resistance. Our findings may contribute to the development of more effective, immune-modulating cancer therapies, thereby improving clinical outcomes for patients with ovarian adenocarcinoma.

## 2. Results

### 2.1. Cell Viability and Inhibitory Concentration

**Figure 1:**
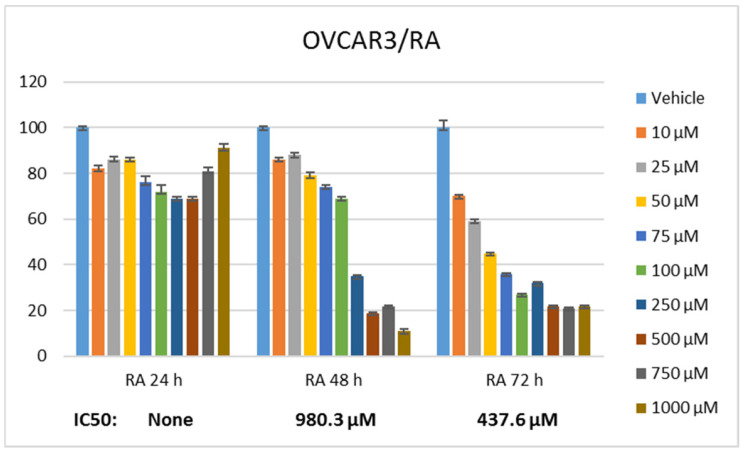
Effect of 24, 48, and 72 h treatment of RA in the concentration range of 10–1000 µM. Inhibition concentration (IC_50_) was calculated by Probit analysis.

**Figure 2:**
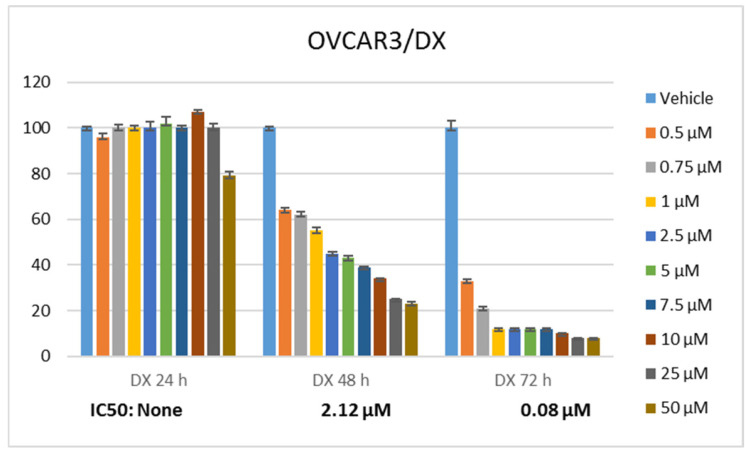
Effect of 24, 48, and 72 h treatment of DX. Inhibition concentration (IC_50_) was calculated by Probit analysis.

Statistically significant differences were determined when the proliferation rate of ovarian cancer cells was compared between RA and DX treatments. It was seen that RA (Figure 1) and DX (Figure 2) inhibited the proliferation of OVCAR3 cells, and the inhibitory effect increased depending on the dose and time. The IC_50_ of RA was measured as none at the 24th hour, 980.3 μM at the 48th hour, and 437.6 μM at the 72nd hour, respectively (Figure 1). The IC_50_ value of DX could not be measured at the 24th hour, but the 48 h IC_50_ was measured as 5.32 μM and the 72 h IC_50_ was measured as 1.23 μM (Figure 2). After 24 h of DX application, statistical significance occurred after a 2.5 uM dose compared to the control group (*p* ≤ 0.05). In 48 and 72 h of DX application, statistical differences occurred compared to the control in all nine different doses (*p* ≤ 0.05).

The therapeutic potential of RA has been demonstrated in many cancers, but there is no study conducted with ovarian cancer cell lines. The cell viability rate was determined by 24, 48, and 72 h of RA (Figure 1) and DX (Figure 2) treatment of human ovarian adenocarcinoma cells. When we look at the results, it was observed that the increase in cell proliferation was very limited at the 24th hour of DX application but showed a concentration- and time-dependent decrease at the 48th and 72nd hours (Figure 2). OVCAR3 cells decreased in the RA-treated group depending on the RA concentration of the cells. Although it initially increased in the 24 h RA treatment, it rapidly lost its vitality depending on the dose and time. Regardless of the treatment duration, it was observed that when RA concentration was applied in 1000 μM/L, cell viability decreased below 60% (Figure 1). At the 72nd hour, cell activity decreased significantly for both RA and DX treatments, regardless of concentration. Therefore, gene and protein analyses in this study were performed according to IC_50_ RA (Figure 1) and IC_50_ DX incubation (Figure 2). After 24 and 48 h of RA application, statistical significance occurred compared to the control after a 10 uM dose (*p* ≤ 0.05). In 72 h of RA application, significant differences occurred compared to the control after a 25 uM dose (*p* ≤ 0.05).

### 2.2. Cell Migration Findings

**Figure 3:**
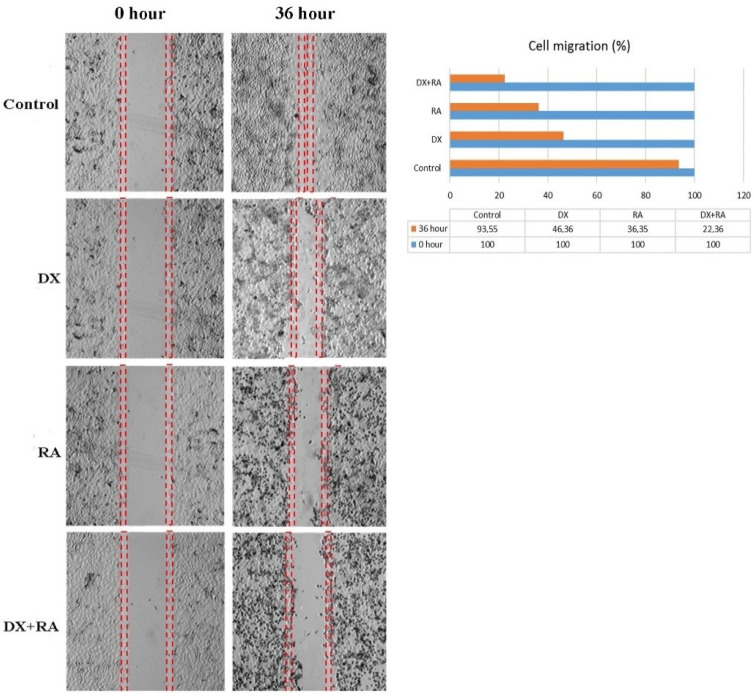
The rate and percentage of wound closure by cell migration after 36 h in the wound healing test in OVCAR-3 ovarian carcinoma cell populations treated with vehicle control, DX, RA, DX+RA IC_50_ (red dashed lines; wound opening borders).

Metastasis rate was determined by wound healing test in OVCAR-3 cells. Time 0 was considered when the wound was created, and the time when control cells completely covered the area was considered as the closure time. Control group cells completely covered the area after 36 h. At the same time, treatment groups were terminated and the area covered by the cells was photographed. Recovery in OVCAR-3 cells was evaluated as % wound size and cell migration compared to the control t = 0. The scratch area closed with nearly 100% cell migration after 36 h in the control group. For the DX treatment group, it was observed that the wound area, which had no cells at hour 0, closed by 46% after 36 h (Figure 3). In the RA treatment group, it was observed that the wound area, which was completely open at hour 0, was closed by 36% cell migration after 36 h. In the DX+RA treatment group, cell migration was found to be 22% at the end of 36 h. Consistent with these results, it was determined that the combination of DX+RA provided higher migration in OVCAR-3 cells than the application of DX and RA alone (Figure 3).

### 2.3. Apoptotic Findings

**Figure 4:**
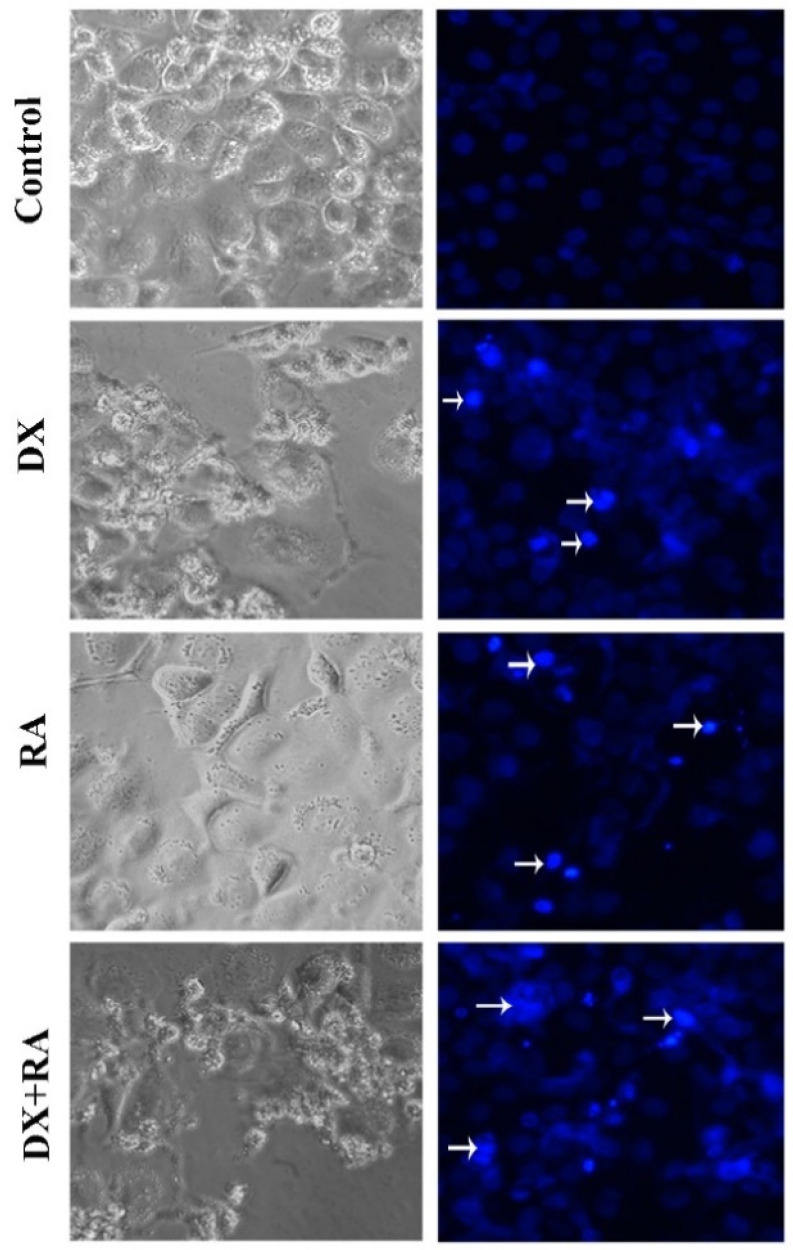
Vehicle control, OVCAR-3 treated with RA IC50, and DX IC50 for 48 h. Cell morphology, nuclear structure, and apoptotic body formation in cell populations (arrow: apoptotic cell, magnification: ×20).

Apoptotic Nucblue staining was performed to determine apoptosis and reveal the apoptotic role of DX and RA. While the morphology and nuclear structure of the cells in the control group appeared normal, it was determined that the cells in the RA and DX treatment groups gained a bright appearance due to the deterioration of the nuclear structure. In this staining, it was seen that the highest apoptotic cell death occurred only in the DX and DX+RA treatment groups (Figure 4).

### 2.4. qRT-PCR (FOXP3, CASP3) Findings

**Figure 5:**
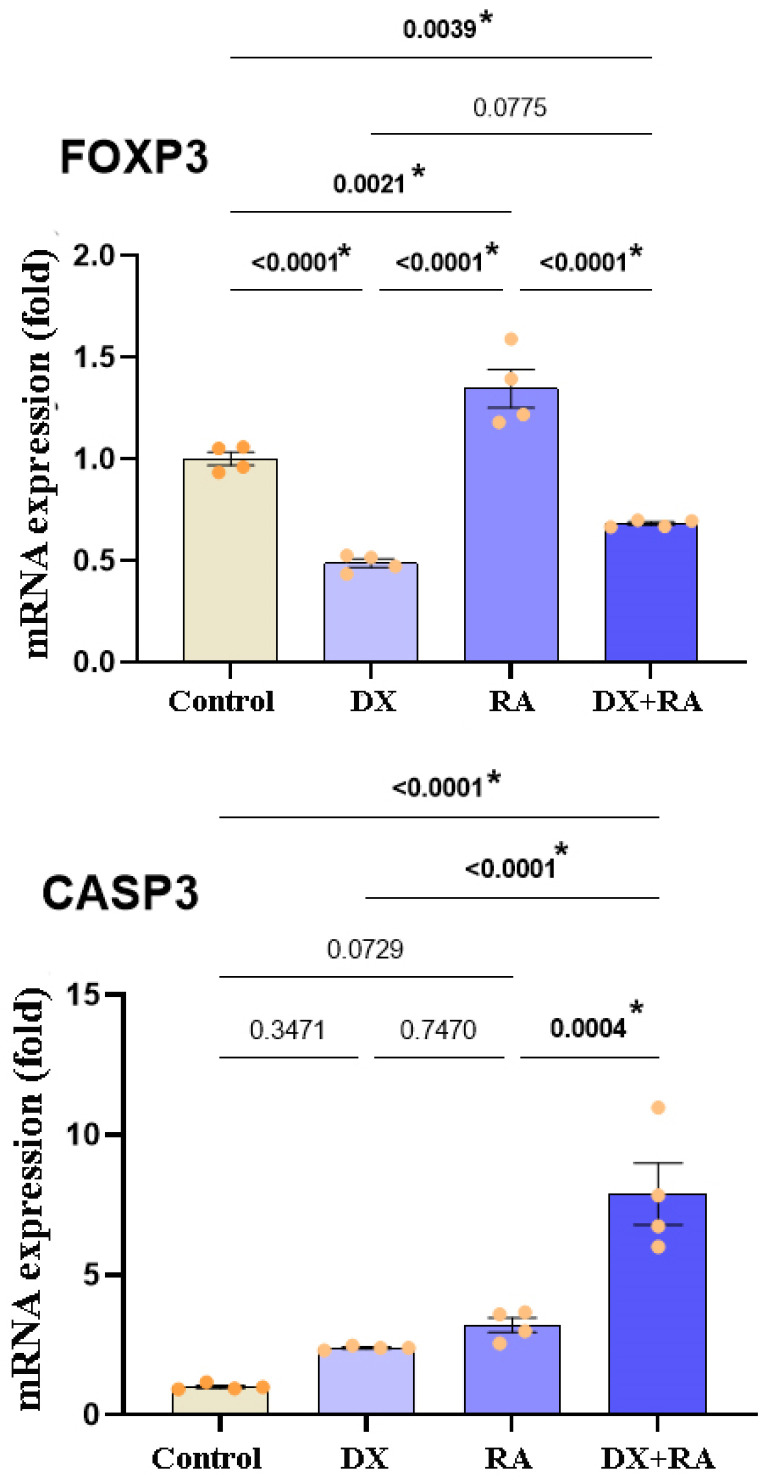
Relative fold values of FOXP3 and CASP3 expressions in OVCAR-3 cell lines (data in multiple control with β-actin and GAPDH mRNA level). Method, n = 4 data mean ± SH). Vehicle control, DX IC_50_: 0.08 µM, and RA IC_50_: 437.6 µM were administered singly and in combination. RNA was isolated from the samples 72 h after the application. * Means are statistically different, and one-way ANOVA, Tukey HSD test, and *p* values are given in the graph.

According to the expression results of genes that suppress the proliferation of cancer cells and drive them to apoptosis, statistically significant differences were detected in FOXP3 and CASP3 gene expressions. When the cells reached the logarithmic phase, vehicle control, DX IC_50_: 0.08 µM, and RA IC_50_: 437.6 µM were administered singly and in combination. The RNA was isolated from the samples 72 h after the application. The statistically significant difference was defined in FOXP3 gene expression between the control and treatment groups. It was observed that gene expression decreased in the control and in the DX and DX+RA groups, and the significance was *p* ≤ 0.0001, and FOXP3 expression was significantly increased in the RA treatment and was significantly different from the control. Additionally, no statistical significance was found between the DX treatment group and the DX+RA treatment group (Figure 5). Again, when the expressions of genes regulating apoptosis were evaluated, CASP3 showed an important increase in all groups compared to the control, while the highest significance was seen only in RA and DX+RA treatments. No statistically significant difference was detected between the control group and the DX and RA treatment groups alone (Figure 5).

### 2.5. PPI Analysis Findings

**Figure 6:**
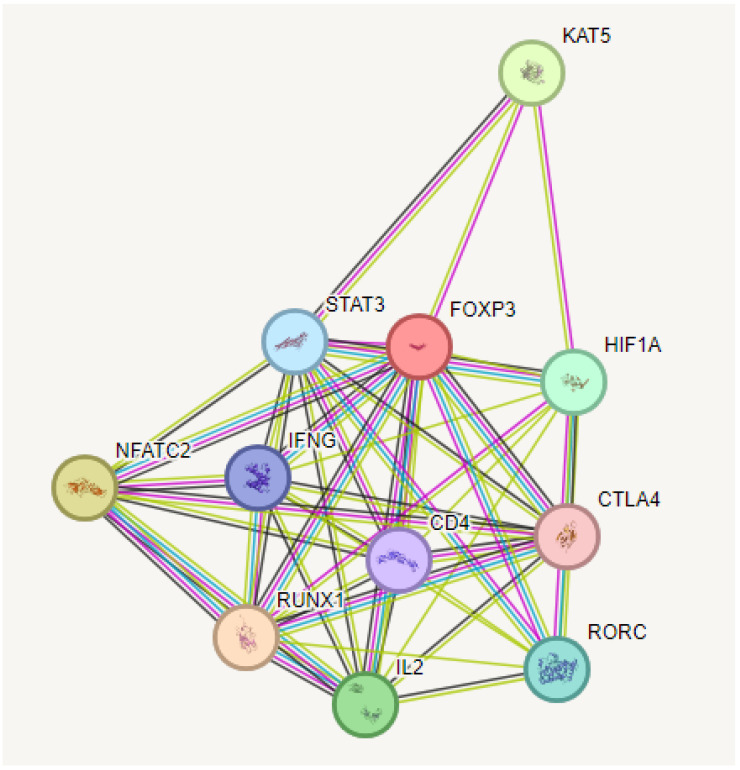
PPI and interaction between various genes of ovarian cancer.

Predictions from STRING analysis were used to depict protein interactions. The visualization showed 11 nodes and 46 edges (Figure 6). Based on nodal degree, the following genes were identified as the top 10 central genes: STAT3, IFNG, NFATC2, RUNX1, IL2, RORC4, CTLA4, HIF1A, CD4, and KAT5. These targets are hypothesized to be the primary targets in ovarian cancer of RA.

### 2.6. KEGG Enrichment Analysis

**Figure 7:**
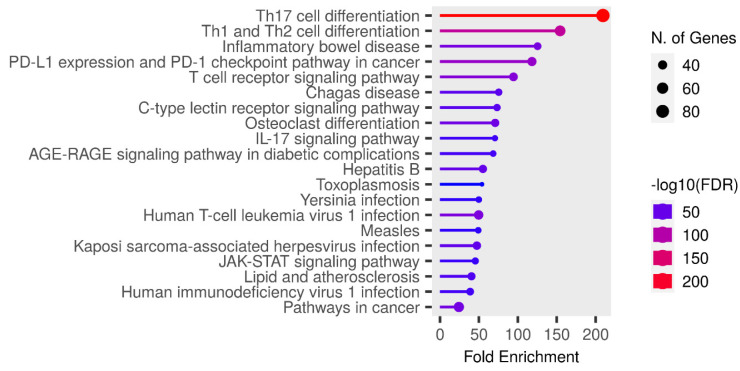
Enrichment analysis for the 90 common compound targets.

KEGG enrichment analysis of the target genes was performed with the Shiny 0.80 program. The findings showed that 90 genes were involved in the enrichment process and 50 pathways were cancer-related, exhibiting a significant correlation with target genes (*p* < 0.05). Basically, Th17 cell differentiation, Th1 and Th2 cell differentiation, inflammatory bowel disease, PD-L1 expression and PD-1 checkpoint pathway in cancer, the T cell receptor signaling pathway, Chagas disease, the C-type lectin receptor signaling pathway, Osteoclast differentiation, the IL-17 signaling pathway, the AGE-RAGE signaling pathway, and the top 10 pathways that occur in diabetic complications are shown (Figure 7).

### 2.7. GO Functional Enrichment Analysis Findings

**Figure 8:**
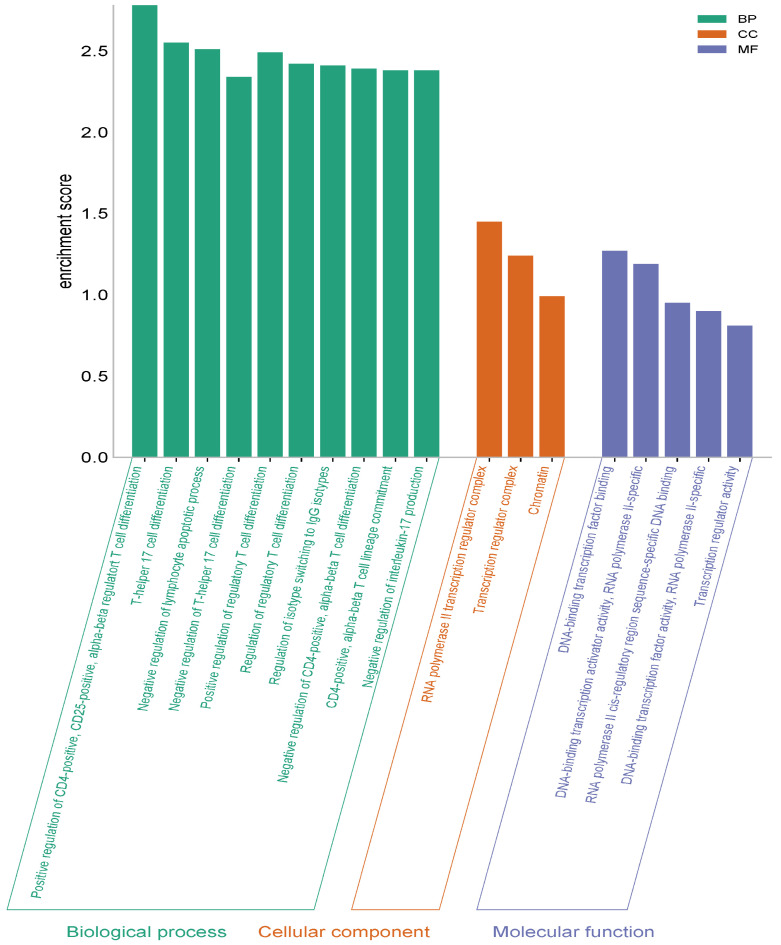
GO (biological process, molecular function, and cellular component) analysis.

The analysis findings show only important functions. The target genes were found to be involved in various cellular components in the BP category, such as helper T cell differentiation and regulation. In terms of cellular components, the target genes have been implicated in the regulation of transcription from the RNA polymerase II promoter, signal transduction, and as a complex regulator of chromatin and transcription. It was found that the MF category exhibited roles such as DNA transcription binding and the binding of RNA polymerase to DNA (Figure 8).

## 3. Discussion

The primary aim of this study was to investigate the modulation of FOXP3 gene expression in OVCAR3 ovarian cancer cells following exposure to rosmarinic acid and doxorubicin. From a molecular perspective, we sought to assess how these agents influence the cellular mechanisms associated with immune regulation and cancer cell survival, specifically through the modulation of FOXP3, a key transcription factor involved in immune suppression. On the genetic level, our objective was to determine whether rosmarinic acid and doxorubicin could alter the expression of FOXP3, potentially impacting the tumor’s ability to evade immune surveillance. By exploring these molecular and genetic pathways, we aimed to provide insights into the therapeutic potential of targeting FOXP3 in combination with chemotherapeutic agents to overcome resistance in ovarian cancer cells.

Since ovarian cancer is diagnosed late, its mortality rate and recurrence are high. It is the major cause of death from gynecological cancer in developed countries [13]. Since it is in an advanced stage when diagnosed, cytoreductive surgery and chemotherapy are preferred for its conventional treatment [13,14,15]. Despite having a very high response rate to treatment, the difficulties in combating it are extremely tiring for the patient, as many of them relapse and develop resistance to chemotherapy [14]. Therefore, early diagnosis of this cancer is very important, and alternative treatments should not be ignored to provide effective treatment. Over the last three decades, numerous preclinical and clinical studies have been conducted with potential drug candidates [16,17].

Considering their strong activities in the fight against cancer, plant-derived compounds have recently gained importance as alternatives to chemotherapy. Promising drugs have begun to be produced for many types of cancer, especially from natural compounds such as RA, curcumin, and alpha lipoic acid [18,19,20]. The main goal is to get rid of the side effects of current chemotherapy. Antioxidants have an important place at this point. Exogenous antioxidants show varying effectiveness in eliminating oxidative stress in vivo. It has been reported that RA increases the expression of anti-inflammatory cytokines IL-6 and FOXP3 and increases the expression of pro-inflammatory cytokines in rats, in which a food allergy model was created with ovalbumin [21]. It has many different advantages, from antioxidant properties to biopharmaceutical properties, from eliminating oxidative stress to absorption at the site of action [20]. The caffeic acid and 3,4-dihydroxyphenyllactic acid found in these plants occur as RA derivatives [22]. There are many studies on the numerous effects of RA, such as antitumor, anti-inflammatory, and antioxidant, and there are reports on its pharmacological effects [23]. Its inhibitory properties have been described in cancers such as skin, pancreatic, and breast cancer [24]. Another study reported that RA develops a mechanism against drug resistance in gastric cancer cell lines [25]. Moreover, it has been proposed that RA is also effective in lung and ovarian cancer and exhibits synergistic effects with chemotherapeutics by increasing their sensitivity [26,27]. The results obtained from studies with RA confirm the anticancer effect on OVCAR3. RA induced apoptosis in ovarian cancer cells and significantly reduced cell viability. It was also able to block proliferation and metastasis, along with DX sensitivity.

In damaged or cancerous tissues, apoptosis allows cells to self-kill and adapt to surrounding tissues, and is also a mechanism that can arise at embryonic development and tumor shrinkage [28]. FOXP3+ regulatory T cells have become therapeutic targets in preventing autoimmune diseases, preventing transplant rejection and inhibiting the progression of tumors, as they have an active mechanism in immune suppression [29]. While one study reported that FOXP3+ T cells could infiltrate tumors [30], another study failed to support these results [31]. It has also been reported that FOXP3+ T cells are mostly found in the peritumoral region [32]. However, another study observed that these cells were located in different locations [33]. Caspase-3 is a member of a conserved family of proteins that is often involved in sustaining apoptosis in cells that are hyper-responsive to specific external or internal stimuli. Furthermore, increasing evidence suggests that caspase-3 plays an important role in the growth and development of malignant cells in multicellular organisms. This study showed that RA inhibited the expression of CASP3, a marker of apoptosis. RA increased CASP3 gene expression, causing apoptosis to be triggered.

Ovarian cancer is diagnosed at an advanced stage, and 70% of patients die before surviving five years. This low survival is due to late diagnosis, abdominal metastasis, and resistance to treatment [34]. The biggest obstacle to its treatment is the ability of cancer cells to evade the immune system [35]. Ovarian cancer is an immunogenic disease and can control immune cell populations around the tumor. One way that ovarian cancer cells evade the antitumor response of the immune system is by attracting suppressive Tregs to the cancerous tissue [36]. FOXP3 plays an important role here, upregulating the expression of Treg-specific genes together with other transcription factors. FOXP3+ Tregs also infiltrate tumors and enable cancer cells to evade the immune system [37]. The best-characterized Tregs are other Treg populations, such as the CD4+FOXP3+ and CD8+ subsets. However, their functions remain unclear. Tregs in ovarian cancer lesions suppress the immune response to tumor-associated antigens, which is achieved by suppressing the secretion of Interferon gamma (IFN-γ) and interleukin-2 (IL-2) by effector T cells. Due to this suppression, Tregs may be an important indicator of prognosis in ovarian cancer patients [36]. In ovarian cancer, Tregs have been shown to support cancer development and resistance at different cancer stages. Understanding the escape mechanisms of these cells offers important opportunities for effective and long-lasting antitumor immunity. Zhang et al. reported that the expression of miR-150-5p and miR-150-3p in ovarian cancer was lower than that in normal ovarian tissues through Gene Expression Omnibus dataset analysis. The study showed that miR-150-5p/3p could inhibit tumor migration and invasion both in vitro and in vivo by regulating insulin receptor substrate-1 and Insulin-Like Growth Factor 1 Receptor (IGF1R). Mechanistic studies found that FOXP3 could bind to the promoter of miR-150-5p/3p. Furthermore, FOXP3-miR-150-IRS1/IGF1R was probably regulated by the PI3K/AKT/mTOR pathway through negative feedback [38].

Comparing our findings with the literature, it appears that RA plays a role as a good ROS scavenger. It may exhibit antioxidant or pro-oxidant properties depending on concentration and redox modulation [39]. At low doses, it protects normal cells while eliminating ROS, while at high doses, it drives cells with impaired functions such as cancer cells to apoptosis and increases cytotoxicity. It was also determined that RA exhibited stronger anticancer properties when combined with the chemotherapeutic agent DX. In this study, we investigated the effects of RA on FOXP3. It showed activity on proliferation, migration, and apoptosis by downregulating and CASP3 upregulating. In addition, it was seen that RA increased the effectiveness of the DX combination in OVCAR-3 cells.

As with the majority of studies, the design of the current study is subject to limitations. The manuscript can be supplemented with additional experiments and markers to illustrate the increase in apoptosis in cancer cells treated with a combination of RA and DX (Western blot data, Annexin V). Today, many targeted drugs are in the research phase. Studies on these promising drugs are still ongoing, and new ones are added to them every day. However, some unexpected side effects occur as targeted agents are used because the receptors and signaling molecules that these agents disable are also used by normal cells when necessary. Thus, the normal cell can sometimes remain within the target. In subsequent studies, the cytotoxicity of RA and DX combination therapy on normal cells should be studied in more detail. In addition, experimental evidence shows that the downregulation of FOXP3 has an important role in apoptosis by activating caspase-3. However, the number of studies and clinical trial examples demonstrating the effects of the FOXP3 signaling pathway on cancer growth and proliferation in ovarian cancer is very limited, and the effects of related molecules/pathways on tumor metastasis remain to be investigated [40]. Additional studies on the effects of RA in animal tumor models are important to evaluate its therapeutic potential in ovarian neoplasia. Further studies will be required to elucidate the mechanistic pathways involved in the apoptosis of tumor cells, their metabolism, and their downstream signaling pathways.

## 4. Materials and Methods

### 4.1. Cell Culture

This study was performed using the human ovarian carcinoma cell line NIH:OVCAR-3 (HTB-161™). OVCAR-3 cells were cultured using RPMI 1640 containing 10% FBS for the medium, and the cells were grown in a sterile incubator at 37 °C and 5% CO_2_. The cells were used starting from the fifth passage, and it was completed at the twentieth passage. In this study, stock solutions of DX and RA agents were prepared using ultrapure Ethanol (Merck, Rahway, NJ, USA). In total, 5 mM stock solutions were prepared for Doxorubicin, and 100 mM stock solutions were prepared for RA. In the applications performed, the final concentration of the vehicle in flasks or plate wells was reduced to 0.1%.

### 4.2. Cell Viability

The ovarian adenocarcinoma cells were cultivated in 96-well culture dishes to determine the IC_50_ doses of RA and DX. The next day, 9 different concentrations of DX in a dose range of 0.5–50 µM and RA in the dose range of 10–1000 µM, obtained as a result of a serial dilution, were applied. In MTT cell viability analysis after 24, 48, and 72 h, anticancer agents and a vehicle control group were seeded in 6 wells. After incubation, cell survival analysis was performed by MTT test. In the first step, a 5 mg/mL “Yellow tetrazolium MTT (3-(4, 5-dimethylthiazolyl-2)-2,5-diphenyltetrazolium bromide)” test solution was added in 20 µL. In the second step, the cells in solution were incubated for 4 h, and the cell medium was completely removed after the incubation. In the third step, ultrapure DMSO (Merck, USA) was added at 200 µL and kept in a dark environment for 4 h. Next, the plates were read spectrophotometrically at wavelengths of 492, 570, and 650 nm. The group treated with the vehicle was taken as the control and the obtained value was taken as 100% viability. IC_50_ values for each cancer cell line and anticancer agents in the control and treatment groups were calculated using the SPSS 20 statistical package program.

### 4.3. Cell Migration

For the wound healing model, OVCAR-3 cells were cultivated with approximately 200,000 cells. After the cells in the wells were 90% confluent, a wound was made on the cells with a sterile micropipette tip of 200 µL volume. After the wound opening, shielded cells were removed by washing with PBS (Gibco, New York, NY, USA). Then, serum-free medium containing vehicle, 72 h IC_50_ value of DX, IC_50_ RA, and IC50 DX+RA agents was added to each well. The cells were imaged using the Thermo EVOS^®^ FL Imaging System (Waltham, MA, USA) at hour 0, and the wound model was checked until the wound in the control was completely closed. This study was terminated at the 36th hour when the wound in the control was 90–100% closed, and the wound healing rates of the groups were photographed. In cell migration analysis, wound opening was measured using ImImage J (1.54 version) software. In a total of 3 wells, 4 measurements were made in the widest and narrowest areas of each well, and a total of 12 data points were used for each group. The value obtained from the control group was taken as 100% cell migration, and comparative rates were determined.

### 4.4. Apoptotic Staining

In OVCAR-3 cells, nuclear morphologies and apoptosis after cell death caused by DX and RA agents were detected with NucBlue^®^ Live ReadyProbes^®^ Reagent (Thermo Scientific, USA)-specific dye. In this context, OVCAR3 were planted with 50,000 cells in each well. The next day, vehicle material, 72 h IC_50_ RA, IC_50_ DX, and IC_50_ DX+RA agents were applied to the wells to form groups. Staining was then performed directly on live cells according to the kit protocol, and the cells were incubated for 30 min. In the next period, the plates were photographed using the Thermo EVOS^®^ FL Imaging System using the bright field and fluorescence mode with a ×20 objective and a DAPI filter.

### 4.5. Gene Expression

The Purelink RNA kit (Thermo, USA) was used for total RNA analysis. Total RNA concentration and purity in the samples were determined using an Optizen NanoQ spectrophotometer (Mecasys, Daejeon, Republic of Korea). cDNA synthesis was performed with a High-Capacity cDNA Kit (Life Technologies, Carlsbad, CA, USA) using 100 ng total RNA. Gene expression levels in cDNAs were measured with a RT-PCR system (PikoReal™, Thermo Scientific) using the SYBR Green/ROX qPCR (Thermo Scientific) kit. Using the threshold cycle (CT) values measured with the device, the gene expression levels of the genes belonging to FOXP3 and CASP3 were calculated as fold change according to the 2-ΔΔCT method. According to this method, the following formulations were used: ΔCT = CT (target gene)-CT (β-actin) Δ(ΔCT) = ΔCT (control group)-ΔCT (treatment group). Fold change = 2-ΔΔCT. The primer sequences used in this study are given below. The specificity of the primers to the target gene was confirmed using the NCBI Primer-BLAST program.

- **FOXP3**: F: GTGGCCCGGATGTGAGAAG, R: GGAGCCCTTGTCGGATGATG
- **CASP3**: F: GGTATTGAGACAGACAGTGG, R: CATGGGATCTGTTTCTTTGC
- **β-Actin**: F: CCTCTGAACCCTAAGGCCAAC, R: TGCCACAGGATTCCATACCC
- **GAPDH**; F: CGGAGTCAACGGATTTGGTCGTAT, R: GCCTTCTCCATGGTGGTGAAGAC

### 4.6. Protein–Protein Interaction (PPI)

PPI data were retrieved from the STRING database. The STRING database provides descriptions of protein–protein interactions (PPIs) as well as confidence intervals for data scores. A confidence score greater than or equal to 0.4 was chosen to construct the interaction network of proteins with target genes.

### 4.7. Enrichment Analysis

Data on the functional annotation of genes and the canonical pathways associated with the strong connections established with these proteins were obtained using the ShinyGO 0.80 program.

### 4.8. Gene Ontologies (GOs)

Three types of Gene Ontologies (GOs) were performed on possible target genes: cellular component (CC), biological process (BP), and molecular function (MF). The SRplot bioinformatics program was used to evaluate these data.

### 4.9. Statistical Analysis

The cell viabilities determined by the MTT and expression values obtained from qRT-PCR data were determined by one-way ANOVA. The groups within which the averages fell were analyzed using the Tukey HSD test. Comparisons between the two groups were determined by sample t or Mann–Whitney U test. Analyses were performed with the SPSS 20 (IBM, Armonk, NY, USA) program, and *p* ≤ 0.05 was used.

## 5. Conclusions

Our study provides compelling evidence that the modulation of FOXP3 gene expression in OVCAR3 cells by rosmarinic acid and doxorubicin represents a promising avenue for enhancing therapeutic strategies against ovarian cancer. The observed alterations in FOXP3 expression suggest that these agents may effectively disrupt immune evasion mechanisms commonly employed by cancer cells, thereby sensitizing them to chemotherapy. By targeting both the molecular and genetic pathways involved in cancer progression and immune regulation, our findings open new possibilities for integrating immune-modulating compounds with conventional chemotherapy to improve treatment outcomes. This research not only deepens our understanding of FOXP3’s role in ovarian cancer, but also offers a potential pathway for overcoming chemoresistance and advancing personalized cancer therapies. It is thought that future studies will be useful to elucidate this pathway.

## Data Availability

All data supporting the findings of this study are available in public databases from PubChem, STRING, and within this paper.

## References

[1] Kuroki L., Guntupalli S.R. (2020). Treatment of Epithelial Ovarian Cancer. BMJ.

[2] Barani M., Bilal M., Sabir F., Rahdar A., Kyzas G.Z. (2021). Nanotechnology in Ovarian Cancer: Diagnosis and Treatment. Life Sci..

[3] Rojas V., Hirshfield K.M., Ganesan S., Rodriguez-Rodriguez L. (2016). Molecular Characterization of Epithelial Ovarian Cancer: Implications for Diagnosis and Treatment. Int. J. Mol. Sci..

[4] Chen S.N., Chang R., Lin L.T., Chern C.U., Tsai H.W., Wen Z.H., Li Y.H., Li C.J., Tsui K.H. (2019). MicroRNA in Ovarian Cancer: Biology, Pathogenesis, and Therapeutic Opportunities. Int. J. Environ. Res. Public Health.

[5] Tarhriz V., Bandehpour M., Dastmalchi S., Ouladsahebmadarek E., Zarredar H., Eyvazi S. (2019). Overview of CD24 as a New Molecular Marker in Ovarian Cancer. J. Cell Physiol..

[6] Moradi S., Fazlali A., Hamedi H. (2018). Microwave-Assisted Hydro-Distillation of Essential Oil from Rosemary: Comparison with Traditional Distillation. Avicenna J. Med. Biotechnol..

[7] Hyun Jin C., Yang H.S., Choi D.S., Byun M.W., Kim W.G., Jeong Y. (2013). Rosmarinic Acid Attenuated SIN-1-induced Cytotoxicity in HepG2 Cells through the HO-1 Induction and Radical Scavenging Activity. Food Sci. Biotechnol..

[8] Furtado R.A., de Araújo F.R., Resende F.A., Cunha W.R., Tavares D.C. (2010). Protective Effect of Rosmarinic acid on V79 Cells Evaluated by the Micronucleus and Comet Assays. J. Appl. Toxicol..

[9] Brummelman J., Pilipow K., Lugli E. (2018). The Single-Cell Phenotypic Identity of Human CD8+ and CD4+ T Cells. Int. Rev. Cell Mol. Biol..

[10] Lu L., Barbi J., Pan F. (2017). The Regulation of Immune Tolerance by FOXP3. Nat. Rev. Immunol..

[11] Plitas G., Rudensky A.Y. (2020). Regulatory T Cells in Cancer. Annu. Rev. Cancer Biol..

[12] Grisham R.N., Hyman D.M., Iyer G. (2014). Targeted Therapies For Treatment of Recurrent Ovarian Cancer. Clin. Adv. Hematol. Oncol..

[13] Alemzadeh E., Allahqoli L., Mazidimoradi A., Alemzadeh E., Ghasemi F., Salehiniya H., Alkatout I. (2024). Deciphering Resistance Mechanisms and Novel Strategies to Overcome Drug Resistance in Ovarian Cancer: A Comprehensive Review. Oncol. Res..

[14] Kossaï M., Leary A., Scoazec J.Y., Genestie C. (2018). Ovarian Cancer: A Heterogeneous Disease. Pathobiology.

[15] Cortez A.J., Tudrej P., Kujawa K.A., Lisowska K.M. (2018). Advances in Ovarian Cancer Therapy. Cancer Chemother. Pharmacol..

[16] Ediriweera M.K., Tennekoon K.H., Samarakoon S.R. (2019). Role of the PI3K/AKT/mTOR Signaling Pathway in Ovarian Cancer: Biological and Therapeutic Significance. Semin. Cancer Biol..

[17] Hitl M., Kladar N., Gavarić N., Božin B. (2021). Rosmarinic Acid-Human Pharmacokinetics and Health Benefits. Planta Med..

[18] Ko Y.H., Kim S.K., Lee S.Y., Jang C.G. (2020). Flavonoids as Therapeutic Candidates for Emotional Disorders Such as Anxiety and Depression. Arch. Pharm. Res..

[19] Huang L., Chen J., Quan J., Xiang D. (2021). Rosmarinic Acid Inhibits Proliferation and Migration, Promotes Apoptosis and Enhances Cisplatin Sensitivity of Melanoma Cells Through Inhibiting ADAM17/EGFR/AKT/GSK3β Axis. Bioengineered.

[20] Attia M., Essa E.A., Zaki R.M., Elkordy A.A. (2020). An Overview of the Antioxidant Effects of Ascorbic Acid and Alpha Lipoic Acid (in Liposomal Forms) as Adjuvant in Cancer Treatment. Antioxidants.

[21] Jia B., Shang J., Zeng H., Wang X., Fang M., Xu L., Liu X., Wu K., Gong Z., Yang Q. (2023). Hepatoprotective Effects of Rosmarinic Acid on Ovalbumin-Induced Intestinal Food Allergy Mouse Model. Molecules.

[22] Kim G.D., Park Y.S., Jin Y.H., Park C.S. (2015). Production and Applications of Rosmarinic Acid and Structurally Related Compounds. Appl. Microbiol. Biotechnol..

[23] Swamy M.K., Sinniah U.R., Ghasemzadeh A. (2018). Anticancer Potential of Rosmarinic Acid and Its Improved Production Through Biotechnological Interventions and Functional Genomics. Appl. Microbiol. Biotechnol..

[24] Messeha S.S., Zarmouh N.O., Asiri A., Soliman K.F.A. (2020). Rosmarinic Acid-Induced Apoptosis and Cell Cycle Arrest in Triple-Negative Breast Cancer Cells. Eur. J. Pharmacol..

[25] Li F.R., Fu Y.Y., Jiang D.H., Wu Z., Zhou Y.J., Guo L., Dong Z.M., Wang Z.Z. (2013). Reversal Effect of Rosmarinic Acid On Multidrug Resistance in SGC7901/Adr cell. J. Asian Nat. Prod. Res..

[26] Liao X.Z., Gao Y., Sun L.L., Liu J.H., Chen H.R., Yu L., Chen Z.Z., Chen W.H., Lin L.Z. (2020). Rosmarinic Acid Reverses Non-Small Cell Lung Cancer Cisplatin Resistance by Activating the MAPK Signaling Pathway. Phytother. Res..

[27] Lešnik S., Furlan V., Bren U. (2021). Rosemary (*Rosmarinus officinalis* L.): Extraction Techniques, Analytical Methods and Health-Promoting Biological Effects. Phytochem. Rev..

[28] Wang X., He Z., Liu H., Yousefi S., Simon H.U. (2016). Neutrophil Necroptosis is Triggered by Ligation of Adhesion Molecules Following GM-CSF priming. J. Immunol..

[29] Liu D., Heij L.R., Czigany Z., Dahl E., Lang S.A., Ulmer T.F., Luedde T., Neumann U.P., Bednarsch J. (2022). The Role of Tumor-Infiltrating Lymphocytes in Cholangiocarcinoma. J. Exp. Clin. Cancer Res..

[30] Goeppert B., Frauenschuh L., Zucknick M., Stenzinger A., Andrulis M., Klauschen F., Joehrens K., Warth A., Renner M., Mehrabi A. (2013). Prognostic Impact of Tumour-Infiltrating Immune Cells on Biliary Tract Cancer. Br. J. Cancer.

[31] Asahi Y., Hatanaka K.C., Hatanaka Y., Kamiyama T., Orimo T., Shimada S., Nagatsu A., Sakamoto Y., Kamachi H., Kobayashi N. (2020). Prognostic Impact of CD8+ T Cell Distribution And its Association with the HLA Class I Expression in Intrahepatic Cholangiocarcinoma. Surg. Today.

[32] Kim H.D., Kim J.H., Ryu Y.M., Kim D., Lee S., Shin J., Hong S.M., Kim K.H., Jung D.H., Song G.W. (2021). Spatial Distribution and Prognostic Implications of Tumor-Infiltrating FoxP3- CD4+ T Cells in Biliary Tract Cancer. Cancer Res. Treat..

[33] Zhou G., Sprengers D., Mancham S., Erkens R., Boor P.P.C., van Beek A.A., Doukas M., Noordam L., Campos Carrascosa L., de Ruiter V. (2019). Reduction of Immunosuppressive Tumor Microenvironment in Cholangiocarcinoma by Ex Vivo Targeting Immune Checkpoint Molecules. J. Hepatol..

[34] Corr B.R., Moroney M., Sheeder J., Eckhardt S.G., Sawyer B., Behbakht K., Diamond J.R. (2020). Survival and Clinical Outcomes of Patients with Ovarian Cancer who were Treated on Phase 1 Clinical Trials. Cancer.

[35] Knutson K.L., Maurer M.J., Preston C.C., Moysich K.B., Goergen K., Hawthorne K.M., Cunningham J.M., Odunsi K., Hartmann L.C., Kalli K.R. (2015). Regulatory T cells, Inherited Variation, and Clinical Outcome in Epithelial Ovarian Cancer. Cancer Immunol. Immunother..

[36] Rodriguez G.M., Galpin K.J.C., McCloskey C.W., Vanderhyden B.C. (2018). The Tumor Microenvironment of Epithelial Ovarian Cancer and Its Influence on Response to Immunotherapy. Cancers.

[37] Paluskievicz C.M., Cao X., Abdi R., Zheng P., Liu Y., Bromberg J.S. (2019). T Regulatory Cells and Priming the Suppressive Tumor Microenvironment. Front. Immunol..

[38] Zhang Q., Zhou X., Wan M., Zeng X., Luo J., Xu Y., Ji L., Zhang J.A., Fan P., Zhong J. (2021). FoxP3-miR-150-5p/3p Suppresses Ovarian Tumorigenesis via an IGF1R/IRS1 Pathway Feedback Loop. Cell Death Dis..

[39] Yue L., Ren Y., Yue Q., Ding Z., Wang K., Zheng T., Chen G., Chen X., Li M., Fan L. (2021). α-Lipoic Acid Targeting PDK1/NRF2 Axis Contributes to the Apoptosis Effect of Lung Cancer Cells. Oxid. Med. Cell Longev..

[40] Sarı U., Zaman F. (2024). Effects of rosmarinic acid and doxorubicine on an ovarian adenocarsinoma cell line (OVCAR3) via the EGFR pathway. Acta Cir. Bras..

